# Treatment of metastatic squamous cell carcinoma arising in sacrococcygeal pilonidal sinus: a case report series

**DOI:** 10.3389/fmed.2023.1248894

**Published:** 2023-09-13

**Authors:** Ainara Soria Rivas, Sonia Bea-Ardebol, Elena Vida Navas, Óscar M. Muñoz-Arrones, Luis Jacobo Cabañas-Montero, Antonio Mena-Mateos, Fernando López-Campos, Sara Corral Moreno, Israel Pérez-Muñoz, Fausto González Lizan, María Sanz Pascual, Juan Jose Serrano Domingo

**Affiliations:** ^1^Medical Oncology Department, Ramon y Cajal University Hospital, Madrid, Spain; ^2^Dermatology Department, Ramon y Cajal University Hospital, Madrid, Spain; ^3^Surgery Department, Ramon y Cajal University Hospital, Madrid, Spain; ^4^Radiation Oncology Department, Ramon y Cajal University Hospital, Madrid, Spain; ^5^Orthopedics and Orthopedic Surgery Department, Ramon y Cajal University Hospital, Madrid, Spain

**Keywords:** pilonidal sinus, squamous cell carcinoma, metastatic, chemotherapy, anti-PD-1, case report

## Abstract

**Background:**

Squamous cell carcinoma (SCC) arising in a sacrococcygeal pilonidal sinus is rare, with cases of metastatic disease being even rarer. Among published cases, almost none have reported on systemic treatment.

**Objective:**

This disease has a poorer prognosis than other forms of cutaneous SCC; therefore, our objective is to shed some light on the treatment of metastatic disease.

**Methods:**

We present a series of nine cases treated at a single center, four of whom received systemic treatment. Additionally, other previously reported cases of metastatic disease are included in an attempt to draw stronger conclusions.

**Results:**

Four patients were treated under several treatment regimens, with a median progression-free survival of only 2 months and two instances of partial response (18%). The best result was achieved with cemiplimab. Across all the cases, there was a trend toward a benefit of the use of systemic treatment (HR 0.41, 95% CI 0.15–1.12, *p* = 0.083; median overall survival 13 vs. 8 months).

**Limitations:**

Limitations include the significant lack of information on previously published cases and the extremely heterogeneous nature of the existing information.

**Conclusion:**

The initial systemic treatment should be an anti-PD-1, as with other SCCs. After progression on anti-PD-1, there is no strong evidence to support the recommendation of a specific treatment or sequence: options include cetuximab and/or chemotherapy (platinum, paclitaxel, 5-fluorouracyl).

## Introduction

Pilonidal sinus (PS) is a common and well-recognized condition that is often complicated by infection. The condition was described by Herbert Mayo in 1833 as a cyst in the sacrococcygeal area with hair inside it (1). Fifty years later, Hodge suggested the term “pilonidal” from two words in Latin: “pilus” (hair) and “nidus” (nest) (2). The condition mainly affects young men (3). Treatment usually consists of surgical excision, and the rate of recurrence is high.

Malignant degeneration is a rare complication (occurring in 0.1% of cases) (4–9) and is observed mainly in cases of chronic, recurrent, and neglected primary pilonidal sinus infection. Squamous cell carcinoma (SCC) is the most frequent form of lesion. Metastatic disease is even rarer, with very few cases published to date.

This article presents a series of cases consisting of nine patients treated at our center, five of whom had metastatic or unresectable disease and received systemic treatment. Our intention is to share our experiences with the aim of establishing better therapeutic strategies.

## Clinical cases

### Case 1

Case 1 was that of a 63-year-old white man with a history of PS diagnosed in the last 30 years, with recurrent episodes of suppuration. The patient was a smoker, but had no other comorbidities. In March 2003, a lesion began to grow (measuring up to 20 cm in diameter), so a computed tomography (CT) scan was performed. Extensive local involvement was observed with sacral invasion, inguinal lymph nodes (LNs), and multiple lung nodes (measuring <1 cm). The LNs were punctured and tested negative for neoplasia (the lung nodes were too small to perform a puncture, but were stable for a year).

Due to extension of the condition and symptomatology, an abdominoperineal resection (APR) with sacral resection was performed in February 2004. The patient was diagnosed with well-differentiated SCC with bone and anal sphincter invasion. The LNs were not affected by the tumor. No complementary treatment was administered.

A local relapse was observed in February 2006. Palliative radiotherapy was proposed, but the patient declined and died on 19 January 2007.

### Case 2

Case 2 was that of a 40-year-old white man with a history of PS, diagnosed in 2003. The patient was a smoker, was obese, and had a history of hepatitis B virus (HBV), hepatitis C virus (HCV), asthma, and chronic obstructive pulmonary disease (COPD). The PS was resected in October 2014, and the patient was diagnosed with well-differentiated SCC with affected margins. The patient was reoperated for wider resection (including the presacral fascia) and intraoperative radiotherapy (16 Gy). No additional treatment was proposed. There was no evidence of relapse at least until March 2022, when the patient was lost to follow-up.

### Case 3

Case 3 was that of a 53-year-old white man with a history of PS, diagnosed ~20 years ago and involving chronic suppuration, without prior surgical treatment. The patient was a smoker without other comorbidities. Due to hyporexia and weight loss of 10 kg in the last year, a biopsy was performed in May 2017. The patient was diagnosed with well-differentiated SCC with local bone involvement based on magnetic resonance imaging (MRI). Due to the extension of the lesion, neoadjuvant radiotherapy was performed between 13 July 2017 and 9 August 2017 (50 Gy). APR with in-bloc resection of the sacrum was performed in October 2017. After surgery, the patient required multiple reinterventions due to ischemia of the flaps, with associated necrosis and extensive debridement. After preparation of a dorsal flap, the patient presented with thrombosis of the basilic vein and the brachial and radial arteries, with associated yeast fungemia. Despite treatment with antifungals, the patient died of septic shock on 15 March 2018.

### Case 4

Case 4 was that of a 69-year-old white man with hypertension and a history of PS, resected in 2007. In March 2018, a lesion began to grow in the sacral area, and a biopsy was performed. The patient was diagnosed with well-differentiated verrucous SCC. At the time of diagnosis, he presented with involvement of the sacrum and the anal sphincter. The patient required intravenous antibiotics due to local infection. Subsequently, APR with in-bloc resection of the sacrum was performed on 21 June 2018, with a dorsal flap and intraoperative radiotherapy (12 Gy).

The patient required reoperation for debridement of necrotic margins. During the postoperative period, he presented with progressive anemia, which progressed to hematemesis. Gastroscopy revealed esophageal and duodenal ulcerations. Upon sudden respiratory deterioration, the patient was intubated and presented massive hemoptysis of unknown origin. He died on 20 July 2018.

### Case 5

Case 5 was that of a 70-year-old white man with a history of PS for an unspecified number of years with recurrent infections, who presented for consultation in May 2021 due to bleeding and worsening of pain. The patient was a smoker with a history of hypertension, COPD, hypercholesterolemia, and grade 1 chronic kidney disease. A biopsy was performed in June 2021, and the patient was diagnosed with well-differentiated verrucous SCC. At the time of diagnosis, he presented with iliofemoral adenopathies and coccygeal bone involvement, observed in a PET scan. As the lesion was considered unresectable, chemo-radiation treatment was administered, consisting of 5-fluorouracyl (1,000 mg/m^2^/day for 4 consecutive days) and cisplatin (40 mg/m^2^; two cycles, every 28 days), plus 58.8 Gy between 19 October 2021 and 21 November 2021. The patient exhibited a partial response, so surgery was proposed. APR with in-bloc resection of the sacrum was performed on 24 May 2022, revealing free margins and no bone or LN involvement. Currently (as of June 2023), there is no evidence of recurrence of the disease.

### Case 6

Case 6 was that of a 57-year-old white man with a history of PS, operated on several times in 1992. The patient was a smoker and had diabetes. Due to bleeding and new local infection, a biopsy was performed, and he was diagnosed in March 2004 with well-differentiated SCC. At the time of diagnosis, the presence of bone involvement meant that the lesion was considered unresectable, so neoadjuvant radiotherapy was administered (50 Gy) in July 2004. Local progression was observed, with invasion of the anal canal in September 2004. The patient received first-line chemotherapy treatment with cisplatin (80 mg/m2) and 5-fluorouracyl (800 mg/m^2^/day, 5 consecutive days) from 10 November 2004 to 2 December 2004. The patient had a prolonged admission due to infection, and local and LN progression were evident in June 2005. A second line of treatment was decided upon, with weekly methotrexate (25 mg/m^2^) from 2 June 2005 to 8 September 2005, but the patient exhibited new local progression. He died in February 2006.

### Case 7

Case 7 was that of a 68-year-old white man with a history of PS since he was 17 years old, operated on several times. The patient was a smoker with a history of hypertension. In February 2011, he presented with sepsis of presacral origin; upon fistulectomy, the patient was diagnosed with well-differentiated SCC. No additional treatment was administered. The patient presented with a local relapse in August 2011, with bone involvement. The lesion was considered unresectable; radiotherapy (66 Gy) was administered, along with weekly cisplatin (40 mg/m^2^) from 1 September 2011 to 25 October 2011. New local progression occurred in January 2012. The patient received weekly cetuximab (250 mg/m^2^) from 24 April 2012 to 4 June 2012, but did not respond to this treatment and died on 14 June 2012.

### Case 8

Case 8 was that of a 54-year-old white man with a history of PS since he was 18 years old, operated on several times. He was a smoker and occasional drinker. The patient presented for consultation in September 2016 due to suppuration and was diagnosed with well-differentiated SCC. At the time of diagnosis, he had unresectable locoregional LN involvement. He received first-line chemotherapy with carboplatin AUC 6 every 21 days plus weekly cetuximab (250 mg/m^2^) from 23 November 2016 to 31 January 2017. The patient exhibited skin response, but bone progression occurred, so second-line radiotherapy treatment (37.5 Gy) was administered, along with a single cycle of mitomycin-C (10 mg/m^2^) and 5-fluorouracyl (1,000 mg/m^2^/day for 4 consecutive days) in March 2017. The patient then presented with LN, skin, and lung progression in April 2017.

He received a third line of treatment with Tegafur (1,000 mg/m^2^/day in three doses) from 1 June 2017 to 4 July 2017, with clinical progression. He was therefore switched to a fourth line of treatment with weekly paclitaxel (80 mg/m^2^) from 20 July 2017 to 29 August 2017. The patient presented further local progression and died on 15 September 2017.

### Case 9

Case 9 was that of a 64-year-old white man with a history of PS, resected when he was 45 years old. The patient presented for consultation in July 2010 due to a mass in the presacral area and was diagnosed with well-differentiated SCC. LN involvement was ruled out by fine-needle aspiration. The lesion was considered unresectable, so treatment with radiotherapy (70 Gy), together with weekly cisplatin (40 mg/m^2^), was administered between 23 August 2010 and 8 August 2010.

In terms of relevant history, the patient was a smoker, was hypertensive, and underwent surgery for bladder carcinoma in January 2017 (with neoadjuvant chemotherapy).

The patient presented with a local relapse of the SCC in the right buttock. Salvage surgery was performed on 31 January 2018, which included fragments of the sacrum (free of disease upon histological inspection). A new local recurrence in May 2018 affected the other buttock, and a new resection was performed. LN involvement was suspected in a PET scan performed in August 2018, and this was confirmed by fine-needle aspiration. The lesion was again considered unresectable, so the patient received first-line treatment with cisplatin 100 mg/m^2^ every 3 weeks between 11 September 2018 and 23 October 2018 (three cycles), resulting in a partial LN response, but with local progression.

**Figure 1:**
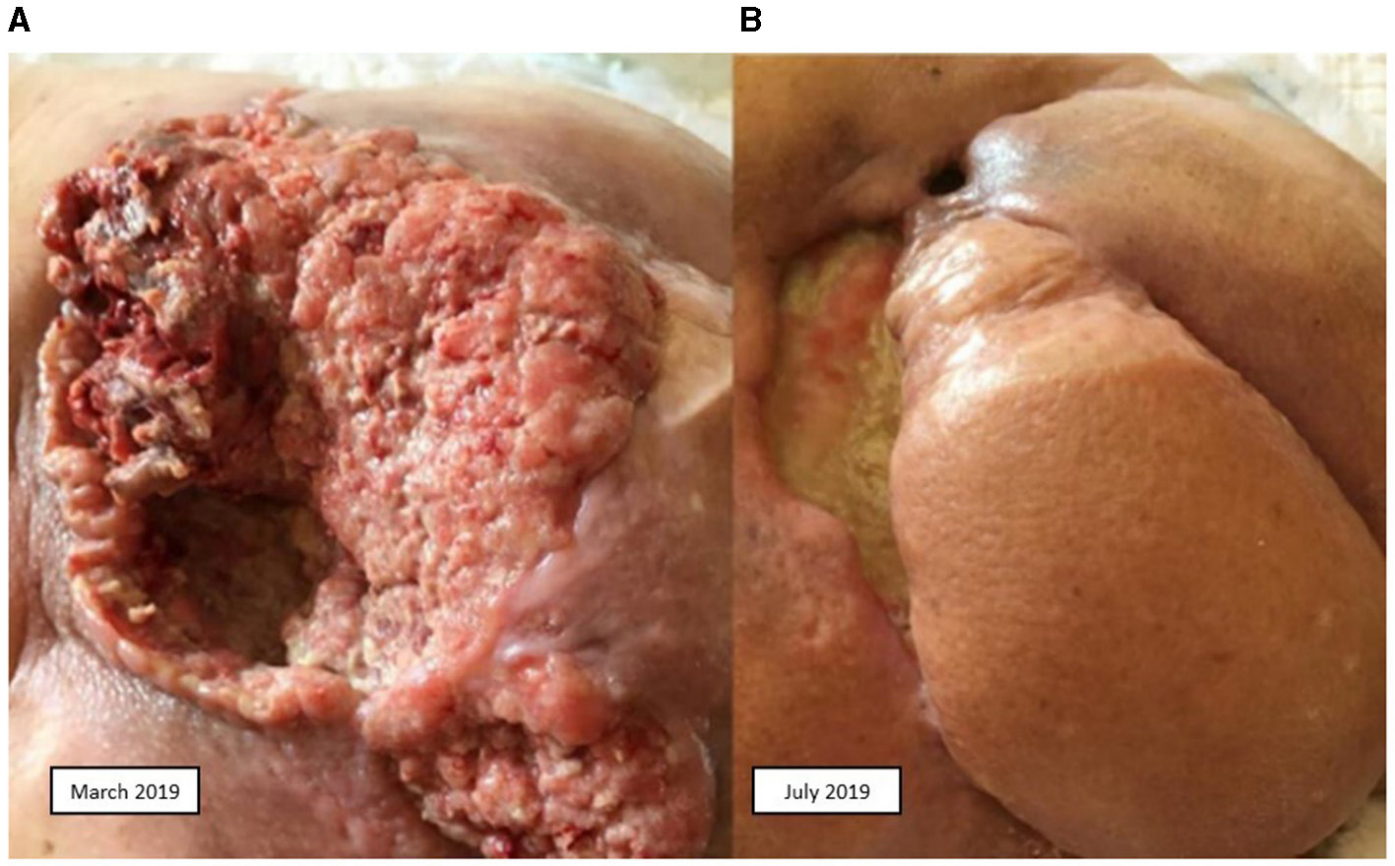
Squamous cell carcinoma. **(A)** Before treatment with cemiplimab. **(B)** After treatment with cemiplimab.

A second line of treatment with biweekly cetuximab (500 mg/m^2^) from 13 November 2018 to 21 February 2019 was decided upon. The patient presented local and nodal progression, so a decision was made to re-irradiate the sacral and LN area with 30 Gy. Upon new progression of the disease, also involving the peritoneum and lungs (Figure 1A), a third line of treatment with cemiplimab was initiated on 3 May 2019, achieving a partial response (Figure 1B). Progression was observed in October 2019, so a fourth line of treatment with weekly paclitaxel plus cetuximab (80 mg/m^2^; 250 mg/m^2^) was initiated on 5 November 2019. A partial response was achieved. This treatment was administered until March 2020, with progression occurring in April 2020, and the patient died on 27 May 2020.

Our cases are summarized in Table 1.

**Table 1:** Clinical cases.

**ID**	**Year (age)^∧^**	Time of PS^*^	**Local invasion**	**Surgery**	**RT**	**Relapse (months)**	**Metastatic sites**	**Systemic treatment**	OS^**^	**Died**
1	2003 (63)	33	No	APR	No	Local (24)	No	-	35	Yes
2	2014 (40)	11	No	WR	Yes^a^ (16 Gy)	No	No	-	89	No
3	2017 (53)	20	Bone	APR	Yes^b^ (50 Gy)	No	No	-	10	Yes
4	2018 (69)	11	Bone, rectal	APR	Yes^a^ (12 Gy)	No	No	-	1	Yes
5	2021 (70)	-	Bone	APR	Yes^c^ (58.5 Gy)	No	No	Yes^c^	24	No
6	2004 (57)	12	Bone	No	Yes^b^ (50 Gy)	Yes, local (2)	No, local LN	Yes^e^	23	Yes
7	2011 (68)	51	No	Simple excision	Yes^d^ (66 Gy)	Yes, local (6)	No, only local	Yes^d, e^	16	Yes
8	2016 (54)	36	LN	No	Yes^d^ (37.5 Gy)	-	Lung, bone, skin	Yes^d, e^	12	Yes
9	2010 (64)	19	No	No	Yes^c^ (70 Gy)	Yes, local (93)^∧∧^	Lung, LN, skin	Yes^c, e^	121	Yes

ID, case identification number; PS, pilonidal sinus; RT, radiotherapy; LN, lymph nodes; APR, abdominoperineal resection; WR, wide resection with presacral fascia; QRT, chemo-radiotherapy.

^∧^All cases of well-differentiated squamous cell carcinoma.

^*^In years.

^**^In months.

^∧∧^At this point, two salvage surgeries were performed before administering systemic treatment.

^a^Intraoperative radiotherapy.

^b^Neoadjuvant radiotherapy.

^c^Neoadjuvant chemo-radiotherapy.

^d^Palliative chemo-radiotherapy.

^e^Palliative chemotherapy.

## Discussion

### Localized disease

Malignant degeneration of PS probably has similar causes to those of other chronic wounds or ulcers [as reported by Marjolin (10)], but the exact mechanism is unknown. In chronic inflammation, normal DNA repair mechanisms are impaired. The process probably begins with the release of free oxygen radicals by activated inflammatory cells, which causes genetic damage and subsequently leads to neoplasia transformation (11). Multiple theories have been proposed to explain this carcinogenic process, and although it is difficult to distinguish primary malignant ulcers from secondary ones, the time course of evolution may help in differentiating them (12).

The most affected patients are men (80%), with a median age of 52 years and a median duration of symptom complaints of 20 years. The most frequent histology is SCC (92%) (13). Other histologies have also been described (14–17).

Before any procedure, it is recommended to perform an exhaustive extension study: computed tomography of the chest, abdomen, and pelvis to rule out distant metastasis (7, 13, 16, 18), and magnetic resonance to determine the local extension. An endoscopic study could be considered to rule out rectal involvement (13, 15, 18). Locoregional LNs may be affected at diagnosis; when this is suspected, a puncture and/or a positron emission tomography (PET) scan should be performed (19).

### Treatment approach

The prevailing treatment approach remains wide excision with margins, including the presacral fascia (6–8, 13, 15, 18), subcutaneous tissue, gluteal muscle, and, if LNs are affected, lymphadenectomy. Prophylactic lymphadenectomy has not been shown to increase survival, although the number of reported cases is too low to draw firm conclusions (11, 20).

In cases of local bone involvement, this can be resected in bloc together with the primary lesion (6–8, 13, 16, 18). APR can also be performed if the rectum is involved (8, 13, 15, 16, 18). Closure of the defect can be achieved with flaps or grafts, or it can be allowed to heal by secondary intention (5, 7, 8, 13, 15, 16, 21).

There is controversy as to whether adjuvant radiotherapy improves prognosis in cases of SCC (22–24). For SCC originating in PS (psSCC), many researchers recommend it, as it has been linked to a reduction in local recurrences from 44 to 30% (8, 13, 15, 18, 21, 25).

Whether the addition of chemotherapy is beneficial remains an unanswered question. In the few published cases (7, 11, 26–28), the drugs used have been 5-fluorouracyl, cisplatin, Adriamycin, and mitomycin-C, in addition to a rare combination without radiotherapy (28).

### Unresectable disease

If upfront surgery is not feasible, treatment with radiotherapy can be considered in conjunction with chemotherapy (13, 25, 27, 29–33). This approach sometimes makes the tumor operable (25).

Cetuximab could also be considered instead of chemotherapy. There are some retrospective studies with other forms of SCC, involving very few patients (median *n* = 8), in which radiotherapy (median dose 60–70 Gy) was administered with weekly cetuximab (34–38). This approach has been found to produce an overall response rate (ORR) of 57–80%, a complete response (CR) rate of 36–75%, and a disease control rate (DCR) of 91–100%. At 2 years, the progression-free survival (PFS) rate has been found to be 50–83% [median PFS (mPFS): 1.6–6.4 months], and the overall survival (OS) rate to be 51–87.5% [median OS (mOS): 3–35 months]. Cetuximab monotherapy could also be considered, as one study has shown that 55.9% of tumors became resectable upon this treatment. Unfortunately, the follow-up duration and the number of patients were excessively low (39).

Another alternative is cryosurgery, based on a series of seven cases with a recurrence rate of 29% and a survival rate of 86%, with at least 7 years of follow-up (40).

The most promising strategy may be neoadjuvant cemiplimab, based on recent data in patients with resectable stage II-IV(M0) SCC, since 51% of such patients achieved CR. However, it was also the case in this study that the median follow-up duration was too short to draw firm conclusions (41).

### Relapses and outcomes

In the case of locoregional relapse, a new resection should be considered, if feasible (6–8). This approach prolongs survival and can even cure the disease (7, 15, 42, 43). Radiotherapy may be considered if it has not been previously administered (9, 44). If surgery is not possible, alternatives include cryosurgery (40), radiotherapy alone (45, 46), and/or systemic treatment, either as a definitive or as a preoperative approach (13, 25).

A 5-year survival rate of 55–61% has been reported (6, 18, 26, 47), representing poorer prognosis compared with other localized SCCs (48, 49).

### Metastatic disease

Metastatic disease is even rarer, with only 22 reported cases (9, 16, 27, 29, 30, 32, 40, 45, 50–56). To these, we add two instances of metastatic cases and two instances of unresectable disease (Table 2).

**Table 2:** Characteristics of metastatic patients.

**Characteristics**	Value^*^
Age: median (range), years	55 (36–68)
Sex: male, *n* (%)	26 (100%)
Median time of PD (range), years	25 (1.5–51)
**Differentiation**, ***n*** **(%)**	
Well-differentiated	10 (63%)
Moderately differentiated	5 (31%)
Poorly differentiated	1 (6%)
**Timing of metastasis**	
Unresectable	2 (8%)
In relapse	18 (69%)
At diagnosis	6 (23%)
**Affected at diagnosis**, ***n*** **(%)**^∧^	
Local nodes	9 (36%)
Bone	8 (40%)
Rectum	2 (10%)
**Treatment at diagnosis**, ***n*** **(%)**	
No treatment	4 (15%)
Surgery	9 (35%)
Surgery + RT	7 (27%)
QT + RT	4 (15%)
RT	1 (4%)
Other	1 (4%)
**Metastatic sites**, ***n*** **(%)**^∧^	
Lung	9 (53%)
Liver	3 (18%)
Bone	6 (35%)
Lymph nodes	8 (47%)
Skin	2 (12%)
**Systemic treatment"**	7 (27%)

PD, pilonidal disease; RT, radiotherapy; QT, chemotherapy.

^*^Numbers do not always sum to 26 due to missing data on some patients.

^∧^The sum is not 100% because options are not mutually exclusive.

^"^Including systemic QT for metastatic patients and QT + RT for unresectable disease or relapses.

**Figure 2:**
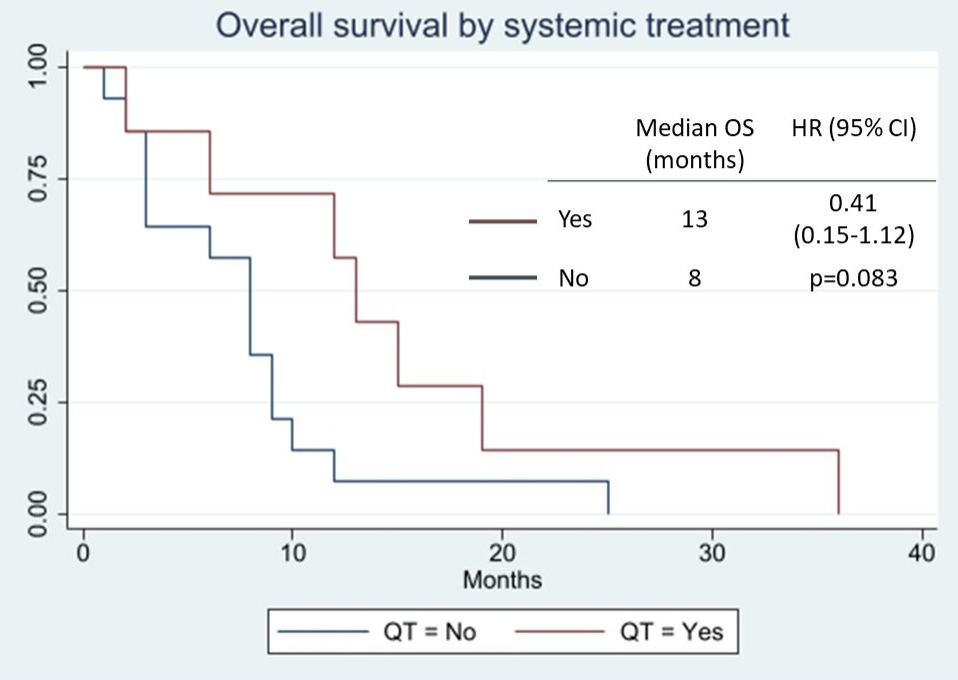
Overall survival by systemic treatment.

In most cases, the OS cannot be inferred, and only two reports have published their chemotherapeutic schedules: one consisting of cisplatin plus 5-fluorouracyl (16) and another consisting of various chemotherapeutic agents (mitomycin-C, vincristine, epirubicin, carboplatin, and 5-fluorouracyl), without mentioning the sequence or whether some drugs were combined (52). No details of either PFS or ORR have been published. At our center, four patients have undergone various treatment regimens. If we group all of the treatments, the mPFS was only 2 months, with two partial responses (18%) (Supplementary Table 1). Again, if we group all the cases in the literature, a trend can be observed toward a benefit of chemotherapy (HR 0.41, 95% CI 0.15–1.12, *p* = 0.083; mOS 13 vs. 8 months; Figure 2).

Treatment choice for SCC has always been a challenge, and regimens have been based on cetuximab and several other chemotherapeutical agents, with platinum, paclitaxel, and fluoropyrimidines being the predominant choices (57–64).

Regarding cetuximab, a phase II study showed a 69% DCR at 6 weeks, ORR of 28% (6% CR), mPFS of 4.1 months, and mOS of 8.1 months (57). Another retrospective study, examining regimens with or without carboplatin, showed similar results: 70% DCR, ORR of 20%, mPFS of 2.65 months, and mOS of 10.35 months. The addition of carboplatin could not be adequately evaluated, but it was not found to be superior (58).

Platinum, in contrast, has produced controversial results when compared with cetuximab. One systematic review showed better results for platinum (mPFS 3.5 vs. 1.9 months; mOS 15.1 vs. 9.8 months) (59), while in another, cetuximab was superior (mPFS 25 vs. 14.6 months; ORR 78 vs. 45%) (60).

Polychemotherapy regimens are less well-tolerated and have not been shown to be better than monotherapy. The largest retrospective study (82 patients) showed an ORR of 18.3% and mOS of 15.3 months. Carboplatin and paclitaxel was the most used combination (61). Cisplatin and 5-fluorouracyl may produce higher response rates, but this regimen is also tolerated less well (62). The addition of bleomycin (63) or anthracyclines (64) has not been shown to achieve better results than cisplatin alone.

It seems that survival is greater in patients who achieve any kind of response than in those who only achieve stabilization. Intralesional methotrexate may be considered for patients with skin lesions that worsen their QoL and who are not suitable for or have exhausted other regimens (65).

However, a revolution in the treatment of SCCs has occurred in the realm of immunotherapy, with findings being reported on cemiplimab in 2018 and on pembrolizumab in 2020 in two phase II studies. Cemiplimab showed an ORR of 47% (CR 7%), estimated progression rate of 53% after 12 months (mPFS not reached), and estimated probability of OS of 81% at 12 months (66). In another study where cemiplimab improved QoL, the results were similar (ORR 46.1% and CR 16.1%), with an incidence of grade 3–5 adverse events of 7.3% (67). Pembrolizumab demonstrated an ORR of 34.3% (CR 3.8%), mPFS of 6.9 months without reaching mOS, and a rate of grade 3–5 adverse events of 5.7% (68).

## Hidradenitis suppurativa

Another entity on which a cutaneous SCC can develop is hidradenitis suppurativa **(**HS), as this is a chronic inflammation. A recent review summarizes 95 cases (69). As in psSCC, the majority of cases are observed in men (77.9%); furthermore, most patients have a long mean time to malignancy (25.5 years) and present mostly well-differentiated histology (62.7%). The most frequently affected areas are the buttocks and the perianal region (47.5 and 18.9%, respectively), and treatment modalities are very heterogeneous. Similarly, this condition also shares the same diagnostic difficulties, usually requiring several biopsies to reach it. The main causes of death are metastases (34.1%) and sepsis (13.6%). Extensive information on systemic treatment is also not available, with a total of 12 patients having received such treatment in different modalities (12.7%). A recent case report has highlighted successful treatment with cemiplimab (70), showing that anti-PD-1 drugs are a credible treatment option for cSCC, regardless of origin.

## Conclusions

Initial management should include computed tomography of the chest, abdomen, and pelvis, as well as an MRI scan. An endoscopic study could be considered to rule out rectal involvement. In the case of suspected involvement of regional LNs, a puncture should be performed. The treatment of choice is surgery with wide margins, including the presacral fascia, with or without resection of the sacrum in bloc. If necessary, APR can be performed. Adjuvant radiotherapy is recommended.

However, due to the high rate of postoperative complications occurring in these cases (three deaths out of six cases of surgery), it might be interesting to consider neoadjuvant treatment (radiotherapy ± chemotherapy; immunotherapy) in the case of large tumors, even if they are resectable.

In cases of unresectable disease, radiotherapy can be administered in combination with chemotherapy (cisplatin with or without 5-fluorouracyl) or cetuximab, although it is possible that the best option may be to assess the use of cemiplimab. If the disease responds to the treatment, resection can be considered. Cryosurgery or intralesional methotrexate are alternatives for frail patients. If local relapse occurs, new surgery should be considered.

In cases of metastatic disease, the absence of studies, the lack of information, and the high levels of heterogeneity among the published cases (including on our part) further complicate decision-making. The most frequently used drugs have been platinum, 5-fluorouracyl, and cetuximab, in a clear attempt to reproduce the results of SCC studies, with little success. However, since this condition is a cSCC, albeit in a different location, we believe that the systemic treatment should be the same as for other forms of cSCC. The proof of this is that the first reported response to systemic treatment in psSCC occurred in one of our patients who received cemiplimab. It should be noted that this response was maintained for 5 months and was observed after the patient had received two other lines of treatment. Subsequently, the same patient presented with a new response to the combination of paclitaxel and cetuximab for another 5 months.

This evidence reinforces the idea that the initial systemic treatment of psSCC should be an anti-PD-1, as in the case of other cSCCs, as established in several clinical guidelines, namely EADO (71), EORTC (71), and NCCN (72). Direct comparisons are lacking, but there exist retrospective studies that have demonstrated an advantage over other systemic therapies (73). After progression to anti-PD-1, there is no strong evidence to recommend a specific treatment or sequence. Options include cetuximab and/or chemotherapy (platinum, paclitaxel, and 5-fluorouracyl).

The lack of information remains a challenge in this condition.

## Patients' perspective

At the time of article submission to the journal, all patients gave consent for publication, with the understanding that this information may be publicly available.

## Data availability statement

The datasets presented in this article are not readily available because of ethical/privacy restrictions. Requests to access the datasets should be directed to the corresponding author.

## Ethics statement

Written informed consent was obtained from the individual(s) for the publication of any potentially identifiable images or data included in this article.

## Author contributions

AS, SB-A, EV, ÓM-A, LC-M, AM-M, FL-C, SC, IP-M, FG, and MS provided the clinical cases from their own experience. AS and JS contributed to conception of the manuscript. JS wrote the first draft of the manuscript. All authors contributed to manuscript revision, read, and approved the submitted version.
