# Supplementary material for: Treatment of metastatic squamous cell carcinoma arising in sacrococcygeal pilonidal sinus: a case report series
Source: Front Med (Lausanne). 2023 Sep 13;10:1248894. doi: 10.3389/fmed.2023.1248894 (PMC10534040; doi:10.3389/fmed.2023.1248894)
Supplement: Supplementary file 1 [file Table_1.DOCX]

Supplementary Table I. Systemic treatment use in our series

| **Treatment** | **ID** | **Number of line** | **Response** | **PFS*** |
| --- | --- | --- | --- | --- |
| Cisplatin + 5-FU | 6 | 1 | No | 7** |
| Methotrexate | 6 | 2 | No | 3 |
| Cetuximab | 7 | 1 | No | 2 |
| Carboplatin + Cetuximab | 8 | 1 | No | 2 |
| Mitomycin-C + 5-FU^ | 8 | 2 | No | 2 |
| Tegafur | 8 | 3 | No | 1 |
| Paclitaxel | 8 | 4 | No | 1 |
| Cisplatin | 9 | 1 | No | 1.5 |
| Cetuximab | 9 | 2 | No | 3 |
| Cemiplimab | 9 | 3 | Yes | 5 |
| Paclitaxel + Cetuximab | 9 | 4 | Yes | 5 |

PFS: progression free survival; 5-FU: 5-fluorouracyl

*In months

**This patient was not evaluated during an income of 3 months, probably, the real PFS was lower

^Plus radiotherapy, 37.5 Grays
